# Rejections in an non-purpose bred assistance dog population: Reasons, consequences and methods for screening

**DOI:** 10.1371/journal.pone.0218339

**Published:** 2019-06-13

**Authors:** Evelien Bogaerts, Christel P. H. Moons, Filip Van Nieuwerburgh, Luc Peelman, Jimmy H. Saunders, Bart J. G. Broeckx

**Affiliations:** 1 Department of Medical Imaging and Small Animal Orthopaedics, Faculty of Veterinary Medicine, Ghent University, Merelbeke, Belgium; 2 Department of Nutrition, Genetics and Ethology, Faculty of Veterinary Medicine, Ghent University, Merelbeke, Belgium; 3 Laboratory of Pharmaceutical Biotechnology, Faculty of Pharmaceutical Sciences, Ghent University, Ghent, Belgium; University of Lincoln, UNITED KINGDOM

## Abstract

Assistance dogs aid people with various impairments on a daily basis. To become an assistance dog, a strict selection procedure and intensive training period must be successfully completed. Consequently, not every dog acquired for this purpose, becomes an assistance dog. The purpose of this study was to investigate reasons for failure and the financial consequences thereof for assistance dog associations that do not have a dedicated breeding program for their dogs. Data were collected for a total of 537 dogs enlisted between 2001 and 2015 and purchased out of the general dog population by five Belgian assistance dog associations. Only 60 percent of the dogs actually became an assistance dog and the main reasons for failure were related to undesirable behavioural characteristics and orthopaedic disorders. The estimated average financial loss per rejected dog was found to be 10524 euro. A detailed comparison of the two most popular breeds (Golden Retriever and Labrador Retriever) within the guide dogs and mobility assistance dogs revealed no significant difference in probability of successfully completing the training. However, a comparison of orthopaedic screening methods revealed a higher rejection with computed tomography for elbow dysplasia and laxity-based radiographical techniques for hip dysplasia compared to radiography and the standard ventrodorsal hip extend radiograph alone, respectively. Based on these results, we provide several suggestions to increase the probability of success.

## Introduction

Assistance dogs are defined as dogs trained to perform tasks for an individual with a disability.[1] All over the world these dogs aid people with various impairments. One example of assistance dogs are the guide dogs (GD) that aid blind or visually impaired handlers during daily activities (e.g. by avoiding obstacles). While GDs are generally well known, they represent only one of the five different subtypes of assistance dogs discussed here.[2] Mobility assistance dogs (MAD) provide practical support to people with mobility impairments by performing tasks like opening doors, retrieving objects that are out of their handlers reach and turning on lights. Hearing dogs aid their hearing impaired or deaf handlers by drawing their handlers’ attention with physical contact upon the occurrence of specific sounds and/or leading them towards the source of the sound. Seizure alerting–and response dogs are trained to alert the handler before the onset of, for example, epileptic seizures or hypoglycaemia or react by warning somebody if a seizure is going on. Autism assistance dogs support children with autism spectrum disorder. Given the wide spectrum of assistance these dogs offer, it is clear that the characteristics of these dogs and the training programs they follow, can differ substantially.[1–3]

Whereas their primary task is to perform according to their specific training, the beneficial effects of these dogs extend much further. They improve the independence of the owner and facilitate social interactions with other people, both leading to enhanced mental and physical well-being.[1,4–7] Additionally, these dogs help to provide a sense of safety, increase the owner’s feelings of competence and offer companionship.[8]

Irrespective of their goal, becoming an assistance dog requires successfully passing a strict selection procedure and an intensive training period. Consequently, many dogs are dropped from the program before being paired with a disabled person. Reasons for failure of dogs are diverse, but often seem to be related to behavioural and orthopaedic phenotypes.[9–12] Most of these studies are based on large assistance dogs associations (ADA) in the United Kingdom and the USA. Frequently, these ADA produce their own puppies in large breeding programs. While breeding programs dedicated to produce assistance dogs optimally suited for the job have been shown to be highly successful, worldwide several, usually smaller, ADA do not have these breeding programs. These organizations usually have to rely on the general dog population to obtain their dogs. To the authors knowledge, literature on these small ADA is lacking however and as such, the knowledge on the reasons for failure, the financial impact and the phases in which these dogs are rejected, is limited.

To fill this gap, we investigated a large group of assistance dogs in Belgium, originating from the general dog population instead of a dedicated breeding program. This research focused on the timing and reasons for rejection, the financial consequences thereof, and the methodology of medical and behavioural screening. Furthermore, we compared the success rates within the two largest groups, i.e. the GD and MAD for the two most common assistance dog breeds (Golden Retriever (GR) and Labrador Retriever (LR)). Based on the results of this study, we provide several suggestions to decrease the number of rejections.

## Methods and materials

### Study design

A retrospective cross-sectional study was performed to collect information on ADA operational aspects and individual dogs.

## ADA operational aspects

A questionnaire was designed to obtain information on dogs that are purchased and trained to become assistance dogs in Belgium. A first part of the questionnaire focussed on general characteristics of the different ADA. General information included the type(s) of assistance dogs trained by the association, average purchase price of a puppy, and the duration and estimated cumulative cost after each of the three different life phases (host family, orthopaedic screening, training). ADA were asked whether they routinely performed screening for medical and/or behavioural traits and, if this was the case, which methodology was used, the age at which screening is performed and which criteria are used when deciding on the suitability of the dog.

### Individual dog information

To allow an overview of the reasons for failure up until the end of the training phase, data were collected on all individual dogs born between 2001 and 2015. The data collected include general demographic variables (date of birth, gender, breed), intended function as assistance dog, the applied screening methods, reasons for rejection of the dog and the timing of when this occurred. Reasons for rejection were grouped in general categories (orthopaedic, internal medicine related diseases, behavioural, neurological, dermatological and the remaining so-called other reasons). ADA were asked to give specific details about the reason for rejection for the orthopaedic and behavioural categories. For orthopaedic reasons, the focus was on elbow dysplasia (ED) and hip dysplasia (HD). In case of a behavioural reason, more details were requested in an open-ended question: “which behavioural issues led to rejection of the dog?”. The information was processed using an inductive thematic analysis, as described in the step-by-step guide by Braun and Clarke.[13] Based on the responses, the following categories of behavioural reasons for drop out were created in the thematic analysis: insecure/anxious, aggression, excitation, motivational issue or prey drive. In case an answer was not relevant to the question, the answer was categorized as invalid.

An overview of the questionnaire is provided in S1 Data.

### Statistics

The statistical analysis can be divided in two major parts. Firstly, descriptive statistics (number of dogs and relative proportions) on reasons for rejection, timing of the rejection (i.e. in which life phase) and the accompanied financial losses were calculated for the entire population. The calculation of the estimated overall financial loss was based on the weighted contribution of each rejection phase and the associated costs for that phase. In a second part, the aim was to compare the overall success rate (dependent variable) between the two most commonly used breeds (the LR and the GR), followed by a more in-depth direct comparison of the two most common reasons for rejection. This comparison was performed with generalized mixed models with breed as fixed effect and ADA as random effect. Finally, a generalized mixed model was used to compare the probability of success (dependent variable) for different screening methods used for orthopaedic disorders (fixed effect; ED: computed tomography (CT) or radiographs; HD: standard hip-extended ventrodorsal (VD) or laxity radiographs) with ADA as random effect. Significance of the fixed effects was each time evaluated with Wald tests and set at α≤0.05. All analyses were performed in R version 3.3.2.

## Results

This retrospective cross-sectional study contains two parts: a general overview of the characteristics of the participating ADA, obtained with a questionnaire, followed by a detailed evaluation of the dogs.

## ADA operational aspects

A total of five Belgian organisations participated in this study. These are relatively small ADA without a breeding program to provide puppies. As a consequence, puppies are purchased from individual dog breeders without a pre-existing purpose to become assistance dog. Two out of these five organisations mainly trained GDs, two others MADs and one trained small amounts of MADs, hearing dogs, seizure alert dogs and autism assistance dogs. Prior to acquisition, puppies were routinely subjected to a behaviour test. Three ADA provided details of their test: they were variations of the Campbell puppy selection test, but the execution and scoring criteria differed between ADA.[14] Irrespective of the purpose of the dog, all organisations followed the same sequence of phases: host family, orthopaedic screening and training with the duration of the different life phases being similar between the different associations. In more detail, the host family phase lasted from eight weeks until 18 months, with one exception where this phase ended at the age of ten months. Most of the dogs were screened for orthopaedic disorders at the age of 12 months. Behavioural screening was, in general, not performed at predetermined ages but rather continuously. The final phase, which is the training phase, took five to ten months and it was at the end of this period that a fully trained dog was paired with a disabled person.

**Fig 1:**
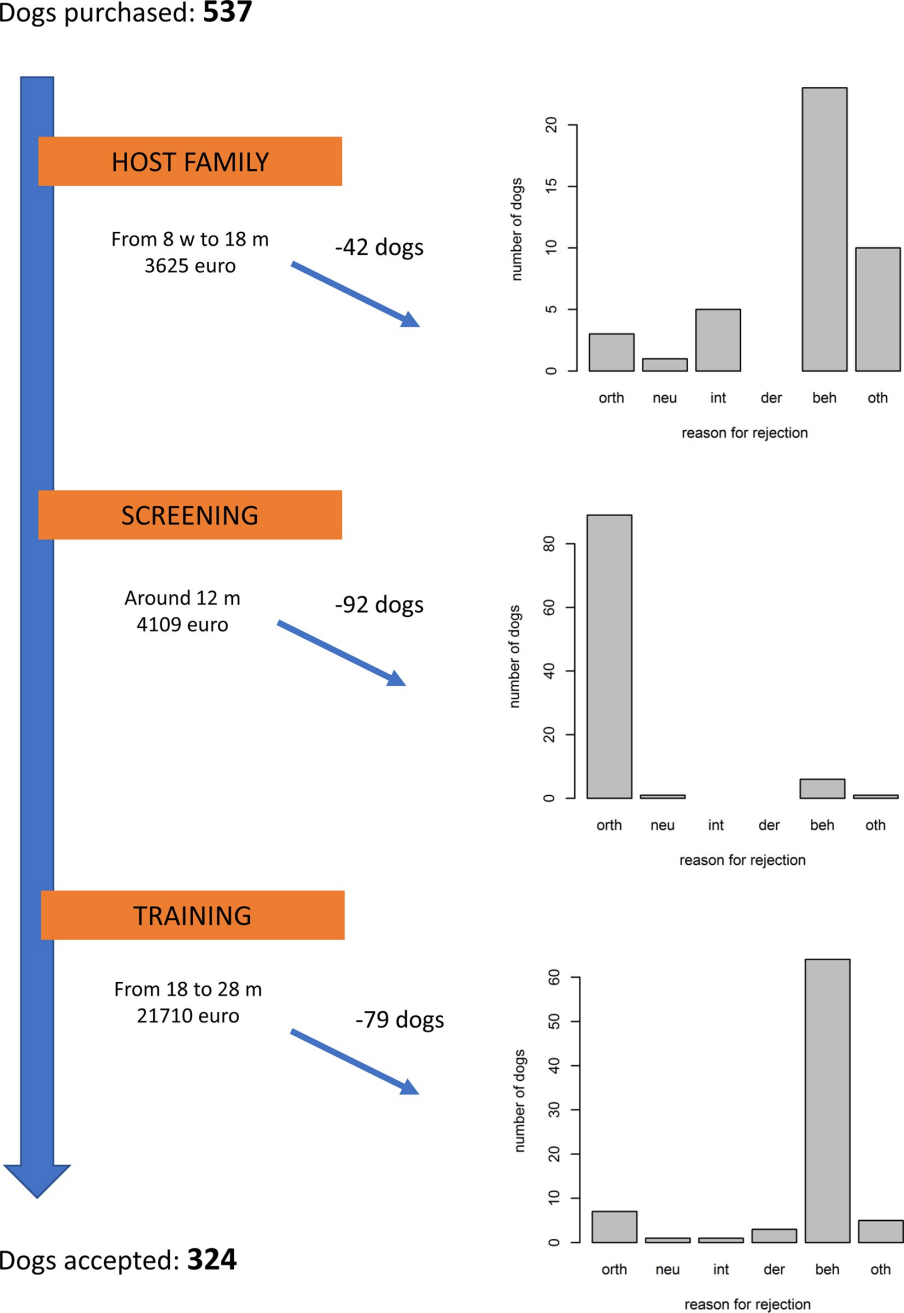
Reason for rejection, number of dogs rejected and estimated cumulative cost per phase. orth: orthopaedic, neu: neurological, int: internal medicine related diseases, derm: dermatological, beh: behaviour, oth: other reasons.

An overview of the estimated cumulative cost in each phase is provided in Fig 1. The average purchase price of a puppy depended on the breed and whether the dog had a pedigree or not. For example, GR and LR puppies with a pedigree (range 1100 to 1300 euro) were on average 600 euro more expensive than dogs without a pedigree (range 500 to 700 euro). The average estimated cost until the end of the host family phase was 3625 euro (range 3500 to 4000 euro), while at the end of the orthopaedic screening the average estimated cost was 4109 euro (range 3984 to 4848 euro). After the training phase, the total cost for a completely trained dog had risen to on average 21710 euro (range from 17500 to 30000 euro). At this stage, the dog had a mean age of 21 months (range from 18 to 24 months).

### Individual dog information

In total, data on 537 dogs were collected. In this population, GDs (234 dogs, 44 percent) and MADs (287 dogs, 53 percent) were represented most, followed by hearing dogs (5 dogs, 1 percent), autism dogs (6 dogs, 1 percent) and seizure alerting–and response dogs (5 dogs, 1 percent). The majority of the dogs were Golden Retrievers (GR) (249 dogs, 46 percent) and Labrador Retrievers (LR) (179 dogs, 33 percent). However, several other breeds were used as well, albeit far less frequent (109 dogs of 21 different breeds, 21 percent, S1 Table).

From these 537 dogs, 92 percent (n = 495) remained at the end of the phase with the host family, while after the orthopaedic screening phase, this decreased to 75 percent (n = 403) and at the end of the training phase, only 60 percent of the originally enlisted dogs (n = 324) actually became assistance dogs. Combining these results with the estimated cost per phase, the average financial loss for a rejected dog was 10524 euro.

Reasons for rejection were grouped in general categories. Eighteen percent of the dogs (n = 99/537) were rejected for orthopaedic disorders and 17 percent (n = 93/537) for undesirable behavioural characteristics. Less reported reasons for rejection were neurological and dermatological disorders (both 0.6 percent, n = 3/537), disorders related to internal medicine (1.1 percent, n = 6/537) and the so-called other reasons (e.g. car accidents; 3 percent, n = 16/537). Seven dogs were rejected for more than one reason (in four dogs the combination of an orthopaedic disorder and a behavioural problem was reported, in two dogs the combination of a dermatological disorder with a behavioural problem and the last dog was rejected for the combination of an orthopaedic–and neurological disorder). In total, this leads to 213 rejected dogs, corresponding to 40 percent of the entire population.

A more detailed evaluation of each phase showed that at the end of the host family phase and during the training phase, most dogs were rejected for behavioural issues, whereas, as expected, the peak rejections due to orthopaedic disorders were during the screening phase (Fig 1). An overview of the orthopaedic screening methods is provided in S2 Data.

Within the group of 99 dogs rejected for orthopaedic disorders, HD was most frequently reported as (one of the) reason(s) for rejection (65 percent, n = 64/99), followed by ED (39 percent, n = 39/99). Importantly, fifteen percent of the dogs rejected for orthopaedic problems were diagnosed with both HD and ED (n = 15/99). Other reasons for rejection were diverse and include tarsus OCD, shoulder OCD, cranial cruciate ligament rupture and fractures (11 percent, n = 11/99).

Overall, 17 percent of the dogs (n = 93/537) were rejected for undesirable behavioural characteristics. Sufficient details for a thematic analysis about why the dog was considered to behave undesirably were available for 73 dogs. There were 63 dogs for which one behavioural reason category was given for their rejection, while two categories were reported for the remaining ten dogs. The most common behavioural reason for rejection was when the dog was found to be insecure and/or anxious, with 41 out of 73 dogs (56 percent) showing this problem. Other issues were aggression to other dogs or humans (22 percent, n = 16/63), excitation (11 percent, n = 8/63), motivational issues (7 percent, n = 5/63), and prey drive (4 percent, n = 3/63).

### Subpopulation of LR and GR within GD’s and MAD’s

The second phase of the statistical analysis examined the two most popular breeds (GR and LR) within the two most trained types of assistance dogs (GD and MAD) (n = 412). Overall, there was no significant difference between the two breeds (Wald test, p = 0.72, Z = -0.35) with respect to the overall success rate to become a fully trained assistance dog. A detailed assessment of the potential reasons for rejection also did not reveal significant differences between the two breeds for any of the main reasons for rejection (orthopaedic (p = 0.96, Z = 0.04) and behaviour (p = 0.65, Z = 0.45)) or for specific orthopaedic diseases (HD (p = 0.23, Z = 1.19), ED (p = 0.46, Z = -0.74)).

### Comparison of orthopaedic screening methods and associated success rates

**Fig 2:**
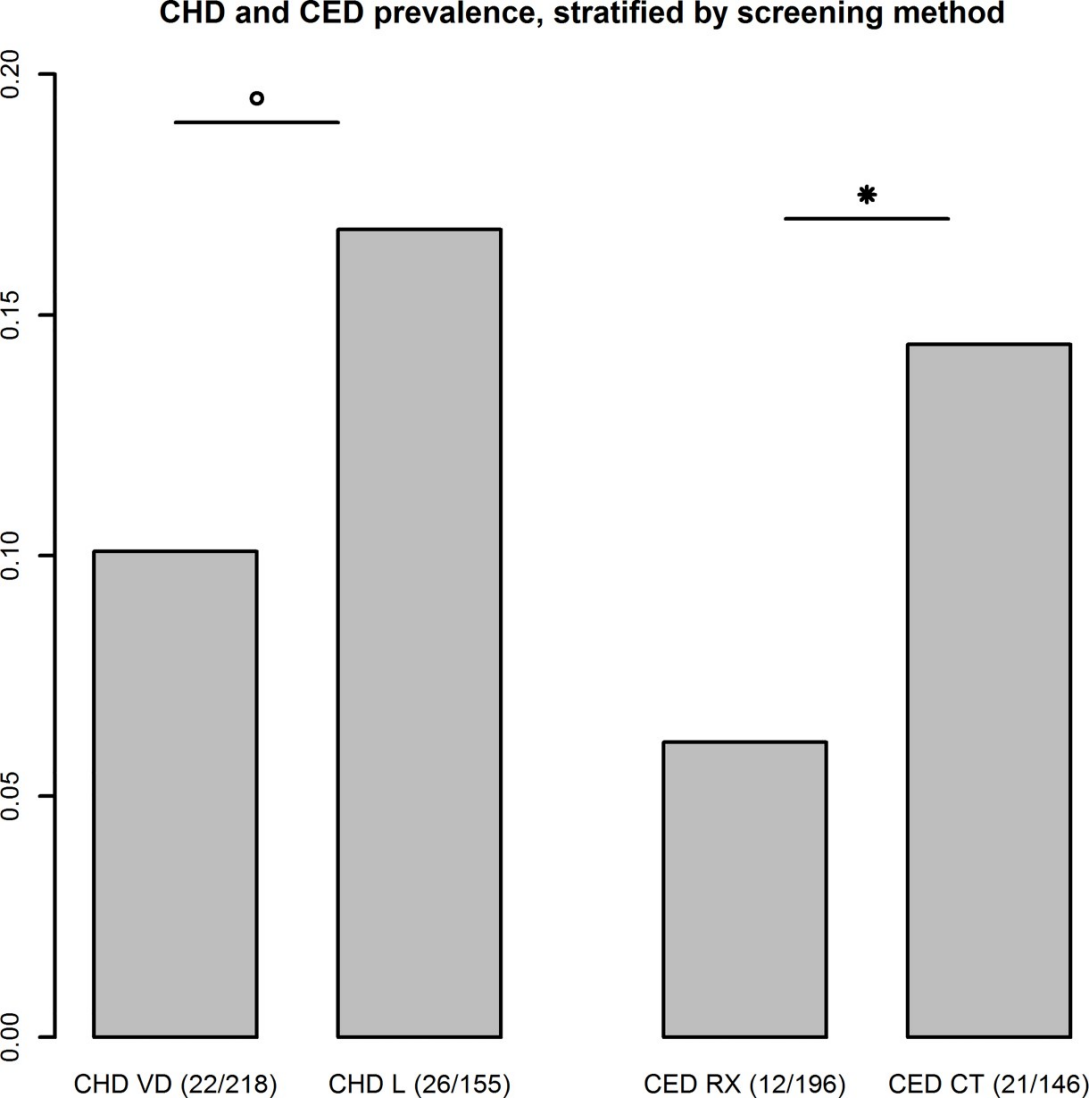
Canine hip dysplasia (CHD) and canine elbow dysplasia (CED), stratified by screening method. VD: standard hip-extended ventrodorsal radiograph, L: laxity-based radiographical technique, RX: radiograph, CT: computed tomography, * and °: significant difference (p ≤ 0.05) between both parameters.

In the early days of assistance dog production and training, only radiography was available for elbow screening while CT has become far more widespread in the latter years. When these two screening methods were compared, it was clear that the probability of failure was far lower with radiography compared to CT (p = 0.01) (Fig 2). In hip joints, a similar trend was noted. Hip joints were evaluated with a VD radiograph, combined with a laxity based technique (PennHip or Vezonni Modified Badertscher distension device technique) in the more recently screened dogs.[15,16] If we looked again at the comparison, less hips were rejected with the conventional VD radiograph compared to a laxity based technique (p = 0.04) (Fig 2).

## Discussion

Based on our results, it is clear that the rejection rate of assistance dogs originating from the general dog population is substantial and has a high financial impact. With a rejection rate of 40 percent, only three out of five dogs complete training successfully and become accepted. Whereas, to our knowledge, no literature is available on assistance dog organizations without a breeding program, rejection rates have been published for some organizations with a breeding program. For example, a study which included five guide and service dog organizations in the USA reported a success rate of 30 to 50 percent.[17] Bray et al (2019) reported a success rate of 43 percent over 13 years in an American organization with different types of assistance dogs, after exclusion for medical reasons and the breeders necessary to maintain a breeding program.[18] At first, this might seem a surprising result as more dogs are rejected than in our study. However, a possible explanation for this higher rejection rate could be a more strict screening protocol within organizations with a breeding program, in combination with higher standards for inclusion of a dog in training and earlier rejection due to the availability of larger numbers of puppies at the start. Where these organizations can actually be very stringent in which dog to select for training, obtaining puppies can be very difficult for organizations without a breeding program, especially if they are small. It has been reported to us that even releasing one or several puppies can have huge consequences for the functioning of these organizations. This might lead to maintaining less suitable dogs in training and even graduating them. Where this might result in a higher number of dogs rejected in their actual working phase, we cannot assess this as this data is not available to us. Overall, it is however clear that rejection rates are substantial.

Overall, the main reasons for rejection are related to orthopaedic disorders (which are predominantly HD and ED) and behaviour. Orthopedically, it is no surprise that both HD and ED account for the majority of rejections. A reason for this high rejection rate due to orthopaedic disorders could be that GR and LR are particularly susceptible to ED and HD.[19,20] ADA however prefer to work with these breeds because of their gentle character, medium stature, trainability and friendly appearance which aids in social interactions.

In terms of behaviour, the high number of rejections might be more surprising at first sight as all puppies that are purchased, are first screened using a behaviour test, in an attempt to predict suitability as an assistance dog.[14] Despite this early screening, 17 percent of the dogs are still rejected for behavioural issues. There are several possible explanations for this. First, scientists have argued and demonstrated that the predictive value of behaviour tests at such a young age is limited, with an additional problem being the lack of test standardization, which was also found in this study.[21–26] Second, performing pre-acquisition screening does not prevent a puppy’s behaviour from being modified by subsequent experiences, which will occur mainly during the host family phase.[25] Although respondents in the survey indicated that obedience is a very important characteristic of a good assistance dog, our results suggest that many dogs have difficulties coping with human society, being insecure/anxious or even showing aggression. According to many studies, anxiety and aggression are also two of the most common behavioural issues in the general pet dog population, although it is not possible to compare the absolute percentages of occurrence, due to differences in definitions and sampling methods.[27,28] Our data indicate that evaluating behavioural functionality should have priority when screening assistance dogs prior to acquisition or during training. This means checking whether puppies are able to cope with contexts where humans are present and with benign human actions, as well as with other stimuli present in a human environment (e.g. specific sounds). If a puppy is anxious in contexts with human presence and fearful of human actions that may or may not be directed towards it, this may indicate that it will not be a suitable working companion. In addition, when a puppy was able to cope during the test, this should still be interpreted with caution as it pertains to the response at that specific point in time. Given the possible influence of environmental factors, more emphasis should also be placed on a careful examination of the puppy socialization program during the host family phase.[29]

It is important to ensure that exposure to social and nonsocial stimuli occurs at an intensity that is pleasant (or at least neutral, depending on the desired result), or that the puppy has control over the stimulus in case the intensity is too high, in order to avoid undesirable learning processes taking place.[30]

As GR and LR are used most often, a direct comparison of these two breeds might reveal breed-specific susceptibilities and, as such, one of the breeds might be more optimal for assistance dogs usage. However, for none of the comparisons, the breeds differed significantly. A study by Duffy and Serpell (2012) could also not demonstrate a difference between LR and GR during estimation of the temperament in young guide- and service dogs done by a C-BARQ evaluation.[31] As such, based on this study and our results, we have insufficient evidence to preferentially select either of the two.

The same population did however reveal an interesting effect of the screening method used to diagnose ED and HD. Over the years, the standard methods for orthopaedic screening went through a striking evolution. Formerly, screening for ED was done by radiography, whereas CT is now used far more often. Taking into account that CT has a higher sensitivity to detect skeletal abnormalities, the higher rejection rate for screening performed with this technique is in agreement with expectations.[32] A similar trend was found for HD, even though HD screening was always performed with radiographs. The main difference for HD is that hip joint screening used to be based on a VD radiograph alone, while in the more recent years, an additional laxity view was added.[15,16] Since the screening of assistance dogs is always performed in young dogs, the majority of dogs will not have developed secondary osteoarthrosis at the moment of screening, while it is also known that this VD radiograph also underperforms when it comes to diagnosing laxity.[33] As such, it is to be expected that adding stress techniques that allow an accurate diagnosis of laxity will lead to an increased detection of dogs with HD.[34] What should be stressed is that the selection of which diagnostic technique was preferably used for the detection of ED and HD was solely based on the availability of the equipment and knowledge and not related to or changed in case of suspicion of the presence an orthopaedic disease. As such, and in agreement with previous literature, it seems to be reconfirmed that modern techniques as CT and stress radiographs have a higher sensitivity to detect certain orthopaedic disorders.

Rejection of a dog to become an assistance dog is always a well-considered and often a difficult decision based on the combination of all medical information and the behaviour. For example, not every dog with radiographic signs of HD will develop lameness in the future.[35,36] With this in mind, it explains why it is very difficult to make the decision to reject or accept dogs with a grade C on a VD radiograph. Luckily, in these cases, the additional laxity based techniques can facilitate the final conclusion, together with the results for elbow screening and behaviour. However, an additional long-term follow-up study to investigate how many of the dogs rejected for orthopaedic issues effectively develop lameness throughout life, might provide a more clear picture on which cut-offs are to be employed.

Based on the findings in this study and a comparison with several highly successful programs worldwide, we see several options to improve the cost-efficiency and reduce the number of rejected dogs. A detailed evaluation demonstrated that the estimated cost increases with every life phase. Especially the training phase is very expensive as this phase is characterized by fulltime training of each individual dog by training staff. A recent financial calculation from Guide Dogs UK is comparable at the level of which life phases are most expensive, although the total cost to train a GD is estimated to be even higher.[37] Due to the huge difference in estimated cost up until the orthopaedic screening phase (4109 euro) versus up until the end of the training phase (21710 euro), it is clear that one possible option to reduce the costs of rejected dogs is earlier release of dogs from the training program. As behaviour is the predominant cause of rejection during the training phase, this practically means that standardized behavioural screening at a suitable age, in combination with an appropriate socialization program during the host family phase should have the largest effect. [38,39] Many assistance dog schools have already attempted to develop validated behaviour tests during the juvenile phase of assistance dogs to predict training outcome and temperament.[18,31,40–44] Implementing these tests into the Belgian program for behavioural screening could possibly lead to earlier detection of behaviour related problems and rejection of unsuitable dogs before the onset of the expensive training phase, if outcome can be accurately predicted.

Changing the breed choice of dogs might also have some effect. Some guide dog schools around the world favor working with GR x LR cross breeds, since they observed a higher training success and reduced withdrawals for orthopaedic disorders.[45,46] Additionally, cross breeds tended to be more likely to have longer healthy lives than purebreds.[31,46,47] Consequently, introducing cross breeds into the Belgian population of assistance dogs could be beneficial to lower the amount of rejections, on both orthopaedic and behavioural level.

There is however a third approach possible if the reasons for rejection are evaluated. Both the orthopaedic disorders (HD and ED) and several behavioural characteristics have been shown to have a genetic basis. [35,48,49] A breeding program could be a possible solution to lower the high amount of rejections in the future. Existing programs already demonstrated worldwide that this genetic selection can be the key to select suitable dogs for the job as assistance dog.[50,51] In more detail, it has for example been demonstrated that 10 generations suffice to get initial hip dysplasia prevalences of 40 to 50 percent to zero. [52] To achieve this genetic progress, a subset of the population of the dogs is selected to be used in breeding programs and this selection can be done based on genomic prediction or estimated breeding values.[53,54] In these breeding programs, other screening criteria can be added when necessary (for example, screening for heart disorders, ophthalmologic screening and genetic tests). Whereas these breeding programs can be a valuable tool, it is however important to emphasize at the same time that they are expensive to implement and maintain and also require a considerable background in quantitative genetics. Furthermore, given the published rejections rates mentioned earlier on, it seems that a direct consequence of having a breeding program is not necessarily lower rejection rates, but more stringent selection criterions.

Overall, there are two limitations in this study. Whereas the main aim was to provide a general overview, it is clear that there are individual differences between the organizations. Given the actual size of these organizations, it is however difficult to compare them individually. Furthermore, it is also clear that thresholds used by the different organizations to reject a dog, can vary. This is especially the case for behavioural characteristics, but also the outcome of some medical screenings can be interpreted subjectively. Even at the level of breeds, behavioural expectations at can differ, leading to different thresholds between breeds as well. It might be interesting for future studies to focus on these individual differences, both at the level of the criteria and rejection rates, while at the same time, the development and implementation of standardized criteria might be an interesting area of research.

## Conclusion

Overall, we conclude that the rejection rate of purchased puppies before matching with a disabled person is high. Only 60 percent of the puppies selected by Belgian organizations actually become an assistance dog. With an average cost of 10524 euro per rejected dog, the subsequent financial loss is substantial. The main reasons for rejection are related to behaviour and orthopaedic disorders. Insecurity and/or anxiety were mainly reported as the behavioural reasons for rejection, while during musculoskeletal screening, HD was the most common reason, followed by ED. While the causes of these high rejection rates seem diverse, we suggest several approaches that can be used individually or combined to improve the success rates.

## Supporting information

S1 Data Questionnaire performed in Belgian assistance dog associations. (DOCX)

S2 Data Screening for orthopaedic disorders. (DOCX)

S1 Table Breed distribution. (DOCX)

## References

[1] Audrestch HM, Whelan CT, Grice D, Asher L, England GCW, Freeman SL. Recognizing the value of assistance dogs in society. Disabil Health J. 2015;8:469–74. 10.1016/j.dhjo.2015.07.001 26364936

[2] Assistance dog international. https://www.assistancedogsinternational.org.

[3] Parenti L, Foreman A, Jean Meade B, Wirth O. A revised taxonomy of assistance animals HHS Public Access. J Rehabil Res Dev. 2013;50(6):745–56. 10.1682/JRRD.2012.11.0216 24203538 PMC4540185

[4] Lane DR, McNicholas J, Collis GM. Dogs for the disabled: Benefits to recipients and welfare of the dog. Appl Anim Behav Sci. 1998;59:49–60.

[5] Rintala DH, Matamoros R, Seitz M. Effects of assistance dogs on persons with mobility or hearing impairements: A pilot study. J Rehabil Res Dev. 2008;45(4):489–504. 18712636 10.1682/jrrd.2007.06.0094

[6] Walther S, Yamamoto M, Thigpen AP, Garcia A, Willits NH, Hart LA. Assistance Dogs: Historic Patterns and Roles of Dogs Placed by ADI or IGDF Accredited Facilities and by Non-Accredited U.S. Facilities. Front Vet Sci. 2017 1 19;4. 10.3389/fvets.2017.00001 PMC5243836 28154816

[7] Hall SS, MacMichael J, Turner A, Mills DS. A survey of the impact of owning a service dog on quality of life for individuals with physical and hearing disability: A pilot study. Health Qual Life Outcomes. 2017;15(59). 10.1186/s12955-017-0640-x PMC5372266 28356121

[8] Clinton R. S. The Impact of Guide Dogs on the Identity of People with Visual Impairments. Anthrozoos. 2000;13(3):131–9.

[9] Goddard ME, Beilharz RG. Genetics of traits which determine the suitability of dogs as guide-dogs for the blind. Appl Anim Ethol. 1983;9:299–315.

[10] Goddard M.E., Beilharz R.G. Genetic and environmental factors affecting the suitability of dogs as guide dogs for the blind. Theor Appl Genet. 1982;62:97–102. 10.1007/BF00293339 24270555

[11] Arata S, Momozawa Y, Takeuchi Y, Mori Y. Important Behavioral Traits for Predicting Guide Dog Qualification. J Vet Med Sci. 2010;72(5):539–45. 10.1292/jvms.09-0512 20009419

[12] Caron-Lormier G, Harvey ND, England GCW, Asher L. A new metric for quantifying the relative impact of risk factors on loss of working life illustrated in a population of working dogs. PLoS One. 2016;11(11):1–17. 10.1371/journal.pone.0165414 PMC5102446 27829045

[13] Braun V, Clarke V. Using thematic analysis in psychology. Qual Res Psychol. 2006;3(2):77–101.

[14] Campbell WE. A behavior test for puppy selection. Mod Vet Pract. 1972;12:29–33.

[15] Smith GK, Bierra DN, Gregor TP. New concepts of coxofemoral joint stability and the development of a clinical stress radiographie method for quantitating hip joint laxity in the dog. JAVMA. 1990;196:59–70. 2295555

[16] Broeckx BJG, Vezzoni A, Bogaerts E, Bertal M, Bosmans T, Stock E, et al Comparison of Three Methods to Quantify Laxity in the Canine Hip Joint. Vet Comp Orthop Traumatol. 2018;31:23–9. 10.3415/VCOT17-05-0064 29325189

[17] Serpell JA, Duffy DL. Aspects of Juvenile and Adolescent Environment Predict Aggression and Fear in 12-Month-Old Guide Dogs. Front Vet Sci. 2016 6 22;3. 10.3389/fvets.2016.00049 PMC4916180 27446937

[18] Bray EE, Levy KM, Kennedy BS, Duffy DL, Serpell JA, MacLean EL. Predictive Models of Assistance Dog Training Outcomes Using the Canine Behavioral Assessment and Research Questionnaire and a Standardized Temperament Evaluation. Front Vet Sci. 2019 2 27;6. 10.3389/fvets.2019.00049 PMC6400848 30873418

[19] Coopman F, Verhoeven G, Saunders J, Duchateau L, Bree H Van. Prevalence of hip dysplasia, elbow dysplasia and humeral head osteochondrosis in dog breeds in Belgium. 2008;136:645–58. 10.1136/vr.163.22.654 19043090

[20] Coopman F, Broeckx B, Verelst E, Deforce D, Saunders J, Duchateau L, et al Combined prevalence of inherited skeletal disorders in dog breeds in Belgium. Vet Comp Orthop Traumatol. 2014;27:395–7. 10.3415/VCOT-13-11-0140 25078710

[21] Beaudet R, Chalifoux A, Dallaire A. Predictive value of activity level and behavioral evaluation on future dominance in puppies. Appl Anim Behav Sci. 1994;40:273–81.

[22] Wilsson E, Sundgren PE. Behaviour test for eight-week old puppies—Heritabilities of tested behaviour traits and its correspondence to later behaviour. Appl Anim Behav Sci. 1998;58:151–62.

[23] Diederich C, Giffroy JM. Behavioural testing in dogs: A review of methodology in search for standardisation. Appl Anim Behav Sci. 2006;97:51–72.

[24] Riemer S, Müller C, Virányi Z, Huber L, Range F. The predictive value of early behavioural assessments in pet dogs—A longitudinal study from neonates to adults. PLoS One. 2014;9(7):e101237 10.1371/journal.pone.0101237 25003341 PMC4086890

[25] Harvey ND, Craigon PJ, Blythe SA, England GCW, Asher L. Social rearing environment influences dog behavioral development. J Vet Behav Clin Appl Res. 2016;16:13–21.

[26] Cobb M, Branson N, McGreevy P, Lill A, Bennett P. The advent of canine performance science: Offering a sustainable future for working dogs. Behav Processes. 2015 1 1;110:96–104. 10.1016/j.beproc.2014.10.012 25444772

[27] Cannas S, Talamonti Z, Mazzola S, Minero M, Picciolini A, Palestrini C. Factors associated with dog behavioral problems referred to a behavior clinic. J Vet Behav. 2018;24:42–7.

[28] Col R, Day C, Phillips CJC. An epidemiological analysis of dog behavior problems presented to an Australian behavior clinic, with associated risk factors. J Vet Behav Clin Appl Res. 2016;15:1–11.

[29] Vaterlaws-Whiteside H, Hartmann A. Improving puppy behavior using a new standardized socialization program. Appl Anim Behav Sci. 2017;197:55–61.

[30] De Keuster T, Monteny J, Moons CP. Behaviour problems: a brief guide in BSAVA Manual of Canine Practice Tim Hutchinson and Ken Robinson, editor. Gloucester, UK: British Small Animal Veterinary Association; 2015. 119–143 p.

[31] Duffy DL, Serpell JA. Predictive validity of a method for evaluating temperament in young guide and service dogs. Appl Anim Behav Sci. 2012;138:99–109.

[32] Rovesti GL, Biasibetti M, Schumacher A, Fabiani M. The use of the computed tomography in the diagnostic protocol of the elbow in the dog: 24 joints. Vet Comp Orthop Traumatol. 2002;15:35–43.

[33] Runge JJ, Kelly SP, Gregor TP, Kotwal S, Smith GK. Distraction index as a risk factor for osteoarthritis associated with hip dysplasia in four large dog breeds. J Small Anim Pract. 2010;51:264–9. 10.1111/j.1748-5827.2010.00937.x 20536696

[34] Smith GK, Paster ER, Powers MY, Lawler DF, Biery DN, Shofer FS, et al Lifelong diet restriction and radiographic evidence of osteoarthritis of the hip joint in dogs. J Am Vet Med Assoc. 2006;5:690–3. 10.2460/javma.229.5.690 16948575

[35] King MD. Etiopathogenesis of Canine Hip Dysplasia, Prevalence, and Genetics. Vet Clin North Am—Small Anim Pract. 2017;47:753–67. 10.1016/j.cvsm.2017.03.001 28460694

[36] Malm S, Fikse F, Egenvall A, Bonnett BN, Gunnarsson L, Hedhammar Å, et al Association between radiographic assessment of hip status and subsequent incidence of veterinary care and mortality related to hip dysplasia in insured Swedish dogs. Prev Vet Med. 2010;93:222–32. 10.1016/j.prevetmed.2009.09.017 19819036

[37] Guide dogs UK [Internet]. Available from: https://www.guidedogs.org.uk/about-us/how-your-money-is-helping/

[38] Asher L, Blythe S, Roberts R, Toothill L, Craigon PJ, Evans KM, et al A standardized behavior test for potential guide dog puppies: Methods and association with subsequent success in guide dog training. J Vet Behav Clin Appl Res. 2013;(8):431–8.

[39] Serpell JA, Hsu Y. Development and validation of a novel method for evaluating behavior and temperament in guide dogs. 2001;72:347–64. 11348683 10.1016/s0168-1591(00)00210-0

[40] Harvey ND, Craigon PJ, Blythe SA, England GCW, Asher L. An evidence-based decision assistance model for predicting training outcome in juvenile guide dogs. PLoS One. 2017;12(6):1–26. 10.1371/journal.pone.0174261 PMC5470660 28614347

[41] Harvey ND, Craigon PJ, Sommerville R, McMillan C, Green M, England GCW, et al Test-retest reliability and predictive validity of a juvenile guide dog behavior test. J Vet Behav Clin Appl Res. 2016;11:65–76.

[42] Bray EE, Sammel MD, Seyfarth RM, Serpell JA, Cheney DL. Temperament and problem solving in a population of adolescent guide dogs. Anim Cogn. 2017 9 1;20(5):923–39. 10.1007/s10071-017-1112-8 28695349

[43] Berns GS, Brooks AM, Spivak M, Levy K. Functional MRI in Awake Dogs Predicts Suitability for Assistance Work. Sci Rep. 2017 3 7;7. 10.1038/srep43704 PMC5339790 28266550

[44] MacLean EL, Hare B. Enhanced Selection of Assistance and Explosive Detection Dogs Using Cognitive Measures. Front Vet Sci. 2018 10 4;5. 10.3389/fvets.2018.00236 PMC6180148 30338264

[45] Ennik I, Liinamo AE, Leighton E, van Arendonk J. Suitability for field service in 4 breeds of guide dogs. J Vet Behav Clin Appl Res. 2006 9;1(2):67–74.

[46] Caron-Lormier G, England GCW, Green MJ, Asher L. Using the incidence and impact of health conditions in guide dogs to investigate healthy ageing in working dogs. Vet J. 2016;207:124–30. 10.1016/j.tvjl.2015.10.046 26616425

[47] Caron-Lormier G, Harvey ND, England GCW, Asher L. Using the incidence and impact of behavioural conditions in guide dogs to investigate patterns in undesirable behaviour in dogs. Sci Reports. 2016;6:23860–23860. 10.1038/srep23860 PMC4831008 27075868

[48] Zapata I, Serpell JA, Alvarez CE. Genetic mapping of canine fear and aggression. BMC Genomics. 2016;17(572). 10.1186/s12864-016-2936-3 PMC4977763 27503363

[49] Lavrijsen ICM, Heuven HCM, Voorhout G, Meij BP, Theyse LFH, Leegwater PAJ, et al Phenotypic and genetic evaluation of elbow dysplasia in Dutch Labrador Retrievers, Golden Retrievers, and Bernese Mountain dogs. Vet J. 2012;193:486–92. 10.1016/j.tvjl.2012.01.001 22336139

[50] Guide Dogs UK [Internet]. Available from: http://www.guidedogs.org.uk

[51] Guiding Eyes for the blind [Internet]. Available from: https://www.guidingeyes.org

[52] Leighton EA, Holle D, Biery DN, Gregor TP, McDonald-Lynch MB, Wallace ML, et al Genetic improvement of hip-extended scores in 3 breeds of guide dogs using estimated breeding values: Notable progress but more improvement is needed. PLoS One. 2019 2 1;14(2). 10.1371/journal.pone.0212544 PMC6386262 30794614

[53] Leighton EA. Genetics of canine hip dysplasia. JAVMA. 1997;210(10):1474–9. 9154200

[54] Soo M. Canine hip dysplasia: phenotypic scoring and the role of estimated breeding value analysis. N Z Vet J. 2015;63(2):69–78. 10.1080/00480169.2014.949893 25072401

