# Supplementary material for: Rejections in an non-purpose bred assistance dog population: Reasons, consequences and methods for screening
Source: PLoS One. 2019 Jun 13;14(6):e0218339. doi: 10.1371/journal.pone.0218339 (PMC6564025; doi:10.1371/journal.pone.0218339)
Supplement: S1 Data — (DOCX) [file pone.0218339.s001.docx]

*Suppl. Data 1*: Questionnaire performed in Belgian assistance dog associations

**Enquête assistentiehonden in België**

1. **Algemene eigenschappen van de vereniging**
   1. Naam van de vereniging
   2. Type assistentiehonden getraind door de organisatie
   3. Gemiddelde aankoopprijs van een puppy
      1. Met stamboom
      2. Zonder stamboom
   4. Wordt er een gedragstest uitgevoerd voorafgaand aan de aanschaf van de pup?
      1. Indien ja, welke test werd uitgevoerd?
   5. Gemiddelde duur van volgende levensfasen:
      1. Fase bij het gastgezin (vanaf welke leeftijd tem welke leeftijd)
      2. Fase van de training van de hond (vanaf welke leeftijd tem welke leeftijd)
   6. Wordt er routinematige medische screening van de hond uitgevoerd?
      1. Welke aandoeningen wordt er gecontroleerd?
      2. Op welke leeftijd?
      3. Welke technieken worden gebruikt?
      4. Criteria die gebruikt worden bij de beslissing om een hond aan te houden of af te keuren
   7. Wordt er routinematige beoordeling van het gedrag van de hond uitgevoerd?
      1. Welke gedragingen worden beoordeeld ?
      2. Op welke leeftijd?
      3. Welke type testen worden gebruikt?
      4. Criteria die gebruikt worden bij de beslissing om een hond aan te houden of af te keuren
   8. Gemiddelde geschatte kost aan het einde van elke levensfase:
      1. Fase bij het gastgezin
      2. Orthopedische screening
      3. Fase van de training van de hond
2. **Individuele eigenschappen per hond**
   1. Algemene informatie
      1. Naam van de hond
      2. Geboortedatum
      3. Ras
      4. Geslacht
   2. Voorziene functie van de assistentiehond
   3. Werd de hond afgekeurd?
      1. Indien ja: tot welke van onderstaande categorieën behoort de reden
         1. Orthopedie
         2. Interne geneeskunde
         3. Gedrag
         4. Neurologie
         5. Dermatologie
         6. Andere
      2. Specifieer voor orthopedie:
         1. Elleboogdysplasie
            1. Werd er screening voor ED uitgevoerd
            2. Welke methode werd gebruikt voor de screening (RX / CT)
            3. Resultaat van de screening
         2. Heupdysplasie
            1. Werd er screening voor HD uitgevoerd
            2. Welke methode werd gebruikt voor de screening (VD radiografische opname / PennHip/ Vezonni Modified Badertscher distention device technique)
            3. Resultaat van de screening
         3. Andere orthopedische aandoeningen
      3. Specifieer voor gedrag:
         1. Welke gedragsmatige problemen hebben geleid tot afkeuren van de hond?
         2. Welke testen werden uitgevoerd om het gedrag in te schatten?
         3. Wanneer werd het gedrag van de hond geëvalueerd?

**Questionnaire assistance dogs in Belgium**

1. **Assistance dog association operational aspects**
   1. Name of the assistance dogs association
   2. Type of assistance dogs trained by the association
   3. Average purchase price of a puppy
      1. With pedigree
      2. Without pedigree
   4. Is any test performed to estimate behaviour prior to acquirement of a puppy?
      1. If yes, which test was performed?
   5. Average duration of following life phases:
      1. Host family phase (age when this phase starts and ends)
      2. Training phase of the dog (age when this phase starts and ends)
   6. Is medical screening performed on a regular basis?
      1. For which diseases do you organize a screening?
      2. At which age is this screening performed?
      3. Which techniques are used ?
      4. Criteria to decide whether to accept or reject a dog
   7. Is behavioural screening performed on a regular basis?
      1. Which behavioural parameters are assessed?
      2. At which age is this performed?
      3. Which kind of tests are used?
      4. Criteria to decide whether to accept or reject a dogs behaviour
   8. Average estimated costs at the end of each life phase:
      1. Host family phase
      2. Orthopaedic screening
      3. Training phase
2. **Individual characteristics per dog**
   1. General information
      1. Name of the dog
      2. Date of birth
      3. Breed
      4. Gender
   2. Intended function as assistance dog
   3. Was this dog rejected?
      1. If yes: which of the following categories was the main reason
         1. Orthopaedic
         2. Internal medicine related diseases
         3. Behaviour
         4. Neurology
         5. Dermatology
         6. Other
      2. Specify for orthopaedics:
         1. Elbow dysplasia
            1. Was screening for ED performed?
            2. Which method was used for screening (RX / CT)?
            3. Result of the screening?
         2. Hip dysplasia
            1. Was screening for HD performed?
            2. Which methode was used for screening (VD radiografische opname / PennHip/ Vezonni Modified Badertscher distention device technique)?
            3. Result of the screening?
         3. Other orthopaedic disorders
      3. Specify for behaviour:
         1. Which behavioural issues led to rejection of the dog?
         2. Which tests were performed to assess the behaviour of the dog?
         3. When was the behaviour of the dog assessed?
