# Supplementary material for: Rejections in an non-purpose bred assistance dog population: Reasons, consequences and methods for screening
Source: PLoS One. 2019 Jun 13;14(6):e0218339. doi: 10.1371/journal.pone.0218339 (PMC6564025; doi:10.1371/journal.pone.0218339)
Supplement: S2 Data — (DOCX) [file pone.0218339.s002.docx]

*Suppl. Data 2:* Screening for orthopaedic disorders

Most of the dogs are screened for orthopaedic disorders between the age of 12 and 22 months and only one association performed the screening twice for the hips with an interval of approximately 14 months (average age at first screening: eight months; average age at second screening: 22 months).

For elbow dysplasia, dogs with the following lesions are removed from training: fragmented medial coronoid process (FMCP), ununited anconeal process (UAP), incomplete ossification of the humeral condyles and/ or presence of secondary osteoarthrosis.

For hip dysplasia, VD radiographs are graded according to the FCI system.[46] In general, dogs with an A and B grade are accepted for training, dogs with a D and E grade are not accepted and dogs with a C grade are in the grey area. For these dogs, a final decision is often made by combining the results for laxity based techniques, elbow- and behavioural screening. Laxity-based diagnostic methods, like PennHIP and a technique employing the Vezzoni modified Badertscher distention device (VMBDD), are used to visualize laxity, which is expressed quantitatively as the distraction- or laxity index, respectively.[15, 16] Dogs with a distraction – or laxity index <0.3 are accepted, dogs with increased laxity in the hip joint are recommended to follow a hydrotherapy program for increasing muscular support of the hip joint and dogs with excessive laxity in the hip joint (>0.7) are rejected.

*References*

15. Smith GK, Bierra DN, Gregor TP. New concepts of coxofemoral joint stability and the development of a clinical stress radiographie method for quantitating hip joint laxity in the dog. JAVMA. 1990;196:59–70.

16. Broeckx BJG, Vezzoni A, Bogaerts E, Bertal M, Bosmans T, Stock E, et al. Comparison of Three Methods to Quantify Laxity in the Canine Hip Joint. Vet Comp Orthop Traumatol. 2018;31:23–9.

46. Soo M. Canine hip dysplasia: phenotypic scoring and the role of estimated breeding value analysis. N Z Vet J. 2015;63(2):69–78.
