# Supplementary material for: Rejections in an non-purpose bred assistance dog population: Reasons, consequences and methods for screening
Source: PLoS One. 2019 Jun 13;14(6):e0218339. doi: 10.1371/journal.pone.0218339 (PMC6564025; doi:10.1371/journal.pone.0218339)
Supplement: S1 Table — (DOCX) [file pone.0218339.s003.docx]

*Suppl. Table 1*: Breed distribution

| American Cocker Spaniel | 1 | Goldendoodle | 4 |
| --- | --- | --- | --- |
| American Staffordshire Terrier | 1 | Golden Retriever | 249 |
| Australian Shepherd | 1 | Labradoodle | 7 |
| Barbet | 1 | Labrador Retriever | 179 |
| Bearded Collie | 1 | Malinois | 1 |
| Beauceron | 1 | Sheltie | 1 |
| Border Collie | 3 | Shiba Inu | 1 |
| Mixed-breed dog | 42 | Standard Poodle | 3 |
| Dachshund | 1 | Tervueren Shepherd | 13 |
| English Cocker Spaniel | 1 | Weimaraner | 1 |
| Flatcoated Retriever | 3 | White Shepherd | 18 |
| German Shepherd | 4 |  |  |
